# Registration, Evaluation, and Authorization of Chemicals (REACH) Is but the First Step–How Far Will It Take Us? Six Further Steps to Improve the European Chemicals Legislation

**DOI:** 10.1289/ehp.0901157

**Published:** 2009-10-13

**Authors:** Christina Rudén, Sven Ove Hansson

**Affiliations:** Department of Philosophy and the History of Technology, Royal Institute of Technology/Kungliga Tekniska Högskolan, Stockholm, Sweden

**Keywords:** chemicals control, classification and labeling, hazard assessment, REACH, regulatory testing

## Abstract

**Objectives:**

In this commentary we analyze how much data will in fact be generated within REACH.

**Conclusions:**

We conclude that the data requirements for many end points still have not been determined but depend on prioritization criteria and waiving practices that will be decided in the years to come. We propose six important steps toward an improved REACH: *a*) Clarify prioritization and waiving criteria. Implement decisions to ensure that sufficient data are obtained to make first hazard assessments of as many substances and end points as possible. *b*) Increase data requirements. Introduce data requirements similar to those currently required for substances produced or imported in quantities of ≥ 10 metric tons/year for substances produced or imported in quantities of ≥ 1 metric tons/year. *c*) Develop the tests and approaches needed to satisfy the information requirements taking into account resource limitations and the aim to reduce animal testing. *d*) Promote substitution of high risk chemicals. Create an effective process for identifying substances of very high concern and for making the appropriate risk management decisions for these substances. *e*) Address the control of substances incorporated in articles. And *f*) acknowledge uncertainties. Systematically report lack of data and include this as a basis for risk management.

After extensive debate and preparation, the new European chemicals legislation, REACH (Registration, Evaluation and Authorisation of Chemicals), was finally adopted in December 2006 (European Commission 2006; includes all REACH annexes described herein). The new legislation aims to improve risk management of industrial chemicals produced in or imported into Europe. Risk management has to be based on a hazard assessment and, if possible, a risk assessment, and the lack of data on the hazards (toxicity and ecotoxicity) of the vast majority of general industrial chemicals was a major justification for the initiative by the European Commission that ultimately led to the new legislation (Allanou et al. 1999; National Research Council 1984; Roe et al. 1997).

REACH came into force on 1 June 2007, and the legislation will be implemented gradually until 31 May 2018. Registration of test data shall be completed before 30 November 2010 for substances produced or imported in quantities of ≥ 1,000 metric tons/year, before 31 May 2013 for substances produced or imported in quantities of ≥ 100 metric tons/year, and before 31 May 2018 for substances produced or imported in quantities of ≥ 1 metric tons/year. (The yearly tonnage refers to metric tons per manufacturer or importer, not to the total volume manufactured or imported.)

The purpose of this commentary is to analyze how much data will in fact be generated within REACH and to relate this to the needs of hazard assessment. We first summarize the data requirements for different tonnage bands, and then relate these requirements to the requirements of hazard assessment. We conclude by proposing improvements in the data requirements and related aspects of the REACH system.

## Data Requirements for the Different Tonnage Bands

According to REACH, chemicals produced or imported in quantities of ≥ 1 metric tons/year (per manufacturer/importer) must be registered in a central database. Unregistered substances may not be manufactured or imported into the European Union in amounts > 1 metric ton/year. In addition to the registration, a technical dossier containing data from whatever tests are mandatory for the substance in question shall be submitted. The data requirements are primarily determined by the produced or imported volume. Substances are divided into five tonnage bands: < 1, ≥ 1, ≥ 10, ≥ 100, and ≥ 1,000 metric tons/year, per manufacturer/importer. In what follows, we summarize the data requirements in each of these tonnage bands. The data requirements accumulate over the tonnage bands, such that for substances produced or imported in quantities of ≥ 10 metric tons/year, the data requirements for substances produced or imported in quantities of ≥ 1 metric tons/year are also applicable, and so on. The data requirements are also summarized in Tables 1–3.

### Substances produced or imported in quantities of < 1 metric ton/year

Substances that are produced or imported in quantities of < 1 metric ton/year by any single manufacturer or importer are not covered by REACH. Obviously, highly toxic or ecotoxic substances produced or imported in these quantities may cause more problems than a less toxic substance produced or imported in much larger volumes. The exclusion from REACH of substances in this tonnage band must be seen as one of the compromises that were made in order to facilitate implementation of the legislation.

### Substances manufactured or imported in quantities of ≥ 1 metric tons/year

The test requirements for the chemicals in this tonnage band are quite complex because these substances are divided into three different categories, as specified in REACH annex VII. The criteria are specified in annex III:

1. Unprioritized phase-in substances are substances that were also regulated in the previous legislation and do not qualify for category 2.
2. Prioritized phase-in substances are substances that were regulated in the previous legislation and in addition fall into one of the following two subcategories (as specified in the REACH annex III): *a*) Substances that are predicted {by the application of (quantitative) structure–activity relationships [(Q)SAR] or other evidence} to be likely to meet the criteria for category 1 or 2 classification for carcinogenicity, mutagenicity, or reproductive toxicity [according to the European classification and labeling directive EEC 67/548; European Economic Community (EEC) 1967], or the criteria for persistent, bioaccumulating, and toxic substances (PBT), or the criteria for very persistent and very bioaccumulating (vPvB) substances (according to annex XIII of REACH). *b*) Substances that both *i*) have dispersive or diffuse (consumer) use(s) and *ii*) are predicted [by the application of (Q)SAR or other evidence] to be likely to meet the classification criteria for any human health or environmental effects end points under the European classification and labeling directive (EEC 1967).
3. Non-phase-in substances are substances introduced on the market subsequent to the REACH implementation.

Only very limited information on physicochemical properties is required for substances in all these three categories: state at 20°C and 101.3 kPa, melting/freezing point, boiling point, relative density, vapor pressure, surface tension, water solubility, octanol/water partition coefficient, flash point, flammability, explosive properties, self-ignition temperature, oxidizing properties, and granulometry.

For unprioritized phase-in substances, no further data are required, and in particular no toxicity testing is required. For non-phase-in and prioritized phase-in substances, the following additional test requirements apply: acute (oral) toxicity, *in vivo* skin sensitization, one *in vitro* test for gene mutations in bacteria (further mutagenicity tests can be required in case of a positive result), acute toxicity to algae and *Daphnia*, and biotic degradation (ready biodegradability) (REACH annex VII). [According to REACH, results from *in vitro* testing of eye and skin irritation are also required for substances produced or imported in quantities of ≥ 1 metric tons/year. However, no such standardized *in vitro* tests are currently available in the Organisation for Economic Co-operation and Development (OECD) test guidelines.]

The actual outcome of the test requirements for the chemicals in this tonnage band will thus depend on how the prioritization criteria are applied. The (Q)SAR models and other methods that will be used to generate data for prioritization purposes have not been defined. A large degree of flexibility is foreseen, and all decisions will be made in collaboration with the industry. According to the REACH guideline,

The process of (Q)SAR acceptance under REACH will involve initial acceptance by industry and subsequent evaluation by the authorities, on a case-by-case basis. It is not foreseen that there will be a formal adoption process, in the same way that test methods are currently adopted in the EU [European Union] and OECD. In other words, it is not foreseen that there will be an official, legally binding list of (Q)SAR methods. With reference to the acceptance criteria in REACH annex XI, it is stated that “the Agency in collaboration with the Commission, Member States and interested parties shall develop and provide guidance in assessing which (Q)SARs will meet these conditions and provide examples.” (European Chemicals Agency 2008)

Because the criteria that will be used to trigger toxicity and ecotoxicity testing in substances in this tonnage band have not yet been decided, it is not possible to foresee how many of these substances will be tested. The outcome of the test requirements for the “phase-in” low-volume chemicals can in principle be that all or none are tested.

### Substances manufactured or imported in quantities of ≥ 10 metric tons/year

For substances produced or imported in quantities of ≥ 10 metric tons per year and per manufacturer or importer, the following additional data are required: *in vivo* skin and eye irritation, acute mammalian toxicity (second route in addition to oral route), acute toxicity to fish and microorganisms (activated sludge respiration inhibition), data on hydrolysis, an adsorption/desorption screening study, and an *in vitro* cytogenicity test using mammalian cells or an *in vitro* micronucleus test. If the mutagenicity tests performed are negative, then an *in vitro* gene mutation study using mammalian cells is also required. If a positive result is obtained in any of the tests, then further *in vivo* mutagenicity studies “shall be considered” (REACH annex VIII). In addition to these tests, a 28-day repeated-dose mammalian toxicity test and screening for reproductive toxicity can be required, but these tests are not mandatory and testing can be waived based on, for instance, the magnitude and nature of human exposures (REACH annex VIII).

The extent to which the 28-day study is waived is important for hazard assessment. If data from a 28-day study are available, then a hazard assessment according to the classification criteria for subchronic toxicity can be made. Without these data, classifications are possible only for acute responses (EEC 1967).

### Substances manufactured or imported in quantities of ≥ 100 metric tons/year and ≥ 1,000 metric tons/year

The ≥ 100 metric tonnage band includes data requirements about fate and behavior (bioaccumulation, simulation testing, and identification of degradation products), long-term toxicity to fish (OECD test guidelines 210, 212, or 215; OECD 1992, 1998, 2000), and *Daphnia*, short-term toxicity to terrestrial organisms and plants, subchronic toxicity to mammals (90 days of exposure), developmental toxicity (OECD test guideline 414; OECD 2001a), and a two-generation reproductive toxicity study (OECD test guideline 416; OECD 2001b) (REACH annex IX). For the ≥ 1,000 metric tonnage band, additional (long-term) effect data on sediment living organisms, terrestrial organisms, and plants can be required, as well as additional data on bird reproduction and a carcinogenicity study (REACH annex X).

In the prefaces to each of these annexes, it is clarified that at this tonnage band “the registrant must submit a proposal and a time schedule for fulfilling the information requirements.” The test requirements are presented in two separate columns, and column 2 lists the “rules according to which the registrant may propose to omit the required standard information, replace it by other information, provide it at a later stage or adapt it in another way.” Furthermore, according to the preface, the registrant “may propose to adapt the required standard information set out in column 1 of this annex according to the general rules contained in annex XI.” Annex XI lists several possibilities to waive testing—for example, based on the exposure scenarios, existing test data, read-across approaches, and (Q)SAR or *in vitro* data, or if there is “sufficient weight of evidence from several independent sources of information leading to the assumption/conclusion that a substance has or has not a particular dangerous property, while the information from each single source alone is regarded insufficient to support this notion.” The major advantage of these rules is that they contribute to avoiding unnecessary testing and introduce flexibility into the system. A possible disadvantage is that the implementation of the test requirements and the outcome of the legislation become less predictable.

Testing in these tonnage bands will thus be proposed by the industry, and the proposal has to be approved by the authority. This applies to all the test requirements in annexes IX and X. Hence, for all the additional tests introduced in the two highest tonnage bands, the actual testing will be determined on a case-by-case basis, and the outcome of test requirements in the high-tonnage bands is thus dependent on how this process is implemented.

## How Much Hazard Assessment Is Possible with the REACH Data?

The purpose of REACH is to generate data on the toxicity and ecotoxicity of industrial chemicals in order to improve risk assessment and risk management. Therefore, it is important to analyze the extent to which the data required by REACH suffice to make a hazard assessment. Of course, ideally a hazard assessment should be based on extensive data that go beyond these data requirements. However, minimal requirements on data, to make a hazard assessment at all meaningful, have been laid down in the European directive for classification and labeling (EEC 1967). That directive does not specify data requirements; it provides the criteria by which available data should be assessed and specifies the corresponding hazard classification categories and the warning labeling that applies to each classification. Hence, whereas REACH lays down the data requirements, the classification and labeling directive regulates how to classify substances according to these data, provide the appropriate hazard information, and convey it to the users of chemicals with the aim to promote safe handling of the substances. Besides the classification criteria, the classification and labeling directive also specifies standardized test methods for different end points. For several end points the directive refers directly or indirectly to a corresponding OECD test guideline. For some end points the preferred methods are not clearly specified (e.g., for mutagenicity testing). However, results from standardized tests—for example, performed according to OECD test guidelines—are readily accepted, and in practice often required, for classification purposes.

The important connection between REACH and the classification and labeling directive makes it important to compare the data required by REACH with the data that are required for hazard assessment of different end points according to the classification and labeling directive. The analyses reported below are based on the criteria as specified according to the European classification and labeling directive (EEC 1967) and its amendments. These criteria will be in force until the year 2015 in parallel with the new globally harmonized system for classification and warning labeling that will then replace this directive (European Commission 2008).

### Acute toxicity

For substances in the 1–10 metric tonnage band that will be categorized either as non-phase-in (i.e., substances introduced on the market after REACH went into force) or as prioritized phase-in substances, the data required in REACH will enable application of the classification criteria for acute (mammalian) toxicity. For the phase-in substances that are not prioritized, no data that can be used for toxicity or ecotoxicity classification will be required by REACH. For substances produced or imported in the 10 metric tonnage band, the data required in REACH will be enough to apply the classification criteria for skin and eye irritation and acute (mammalian) toxicity. This is one of the most important open issues in the implementation of REACH, because classification according to irritation and acute toxicity is fundamental for the safe handling of chemicals, especially in the workplace.

### Subacute toxicity

For substances produced or imported in quantities of < 10 metric tons/year, the data required by REACH will not be sufficient for classification according to the criteria for subacute toxicity (European Commission 2001). For substances produced or imported in quantities of 10–100 metric tons/year, the data required in REACH may suffice to apply these classification criteria. However, this is among the data requirements that can be waived. Decisions yet to be made will thus determine to what extent the requirement of the 28-day study will be implemented or waived. For substances produced or imported in quantities of ≥ 100 metric tons/year, data from the 28-day study will be mandatory.

### Mutagenicity

REACH introduces a tiered approach to mutagenicity testing, which means that the ultimate test requirements are determined based on the results from initial testing in a stepwise procedure. According to the classification and labeling directive, classification as a mutagenic compound can be based on either human or animal data. Human data are required for a category 1 classification (substances known to be mutagenic to humans), but for a classification as a category 2 or 3 mutagen, animal data are sufficient. The criteria are applicable only to *in vivo* data, but apart from that no further specification on appropriate methods is provided. This should be determined on a case-by-case basis (European Commission 2001). Hence, a hazard assessment will be possible only to the extent that positive results are obtained by *in vitro* tests initially required by REACH and, as a consequence, further *in vivo* testing is proposed and performed.

### Carcinogenicity

According to the classification and labeling directive, classification as a carcinogen can be based on either human or animal data. Human data are required for a category 1 classification (substances known to be carcinogenic to humans), but for a classification as a category 2 or 3 carcinogen, animal data are sufficient. In practice, this usually requires data obtained from a standardized long-term carcinogenicity study (European Commission 2001). For substances produced or imported in quantities of < 1,000 metric tons/year, the data required in REACH will not suffice to apply the classification criteria for carcinogenicity. For substances produced or imported in quantities of ≥ 1,000 metric tons/year, such data can be required within REACH, but this requirement can also be waived. Again, the extent to which this requirement will be implemented depends on decisions that are yet to be made (see also Scheringer et al. 2006).

### Reproductive toxicity

The test methods applicable to the classification criteria for reproductive toxicity are not specified in the classification and labeling directive (European Commission 2001). However, in practice, the prenatal developmental toxicity study (OECD test guideline 414; OECD 2001a) is needed to apply the criteria for developmental toxicity, and the two-generation reproduction toxicity study (OECD test guideline 416; OECD 2001b) is needed for classification of effects on fertility (Ohlsson A, Swedish Chemicals Agency, personal communication). The reproductive toxicity screening test that may be required by REACH for chemicals in the ≥ 10 metric tonnage bands is thus usually not sufficient for classification. A hazard assessment for reproductive toxicity according to the classification and labeling criteria will hence be possible only with the REACH data required for substances in the ≥ 100 metric tonnage bands and only to the extent that these test requirements are proposed and approved by the industry and the authority.

### Ecotoxicity

Classification of aquatic toxicity requires, at a minimum, short-term toxicity data from fish, algae, or *Daphnia*. For classification of long-term effects, these data must be used in combination with degradation data (ready biodegradability test), data on lipophilicity (log *P*), or data on bioconcentration (BCF) (European Commission 2001). A hazard classification for aquatic toxicity will thus be possible for substances in the ≥ 10 metric tonnage bands. Whether this will also be possible for the ≥ 1 metric tonnage band depends on the outcome of the REACH prioritization procedures as described above.

### PBT and vPvB

The criteria for identifying PBT and vPvB substances are specified in REACH annex XIII and not in the classification and labeling directive. Application of these criteria will be possible only for substances in the ≥ 100 metric tonnage bands and only to the extent that the tests in the high-tonnage bands will be proposed and approved by industry and the authority. (The criteria are based on BCF, long-term toxicity, or ecotoxicity tests and the half-lives of the substances in different compartments. To determine half-lives, simulation testing is needed.)

Hence, for each of these end points, it is still undetermined for which substances the data minimally necessary for a hazard assessment will be required in the REACH system.

## REACH Was the First Step—What Are the Next Ones?

REACH has provided a structure in which well-informed chemicals risk management can be developed. In particular, it creates a legislative and regulatory framework in which the procurement of data for reasonably reliable hazard assessments is possible. But on the other hand, as we have shown above, it does not require the creation of such data for all substances for which it is needed, and there are important open issues concerning what data will actually be required.

This is not surprising because the deficiencies in the previous system of chemicals regulation were so large that it would be unrealistic to believe that they could be solved in one single reform. It is expected that there is still room for improvement, and the legislation stipulates that the European Commission should, every 5 years, publish a general report on the experience acquired with the operation of the regulation. Furthermore, the REACH process has triggered extensive discussions about what types of testing and how much testing are indeed necessary to characterize hazards and, even more important, to ultimately make science-based risk decisions. This discussion includes developing novel approaches to generating relevant information and principles on how to combine different tests into integrated test systems and how to manage remaining uncertainties and lack of data (e.g., Schaafsma et al. 2009).

## Conclusion

We propose six improvements of the legislation that should be important future steps in the development of REACH.

### a) Clarify prioritization and waiving criteria

The open issues concerning prioritization and waiving practices need to be solved. As much as possible, they should be solved in a way that ensures the availability of the data minimally required to make hazard assessments according to the classification and labeling directive (EEC 1967) for as many substances and end points as possible. The most important of these open issues concerns the prioritization of phase-in substances. For the prioritized phase-in substances, the most basic end points for an elementary assessment of toxicity and ecotoxicity will be covered, but for the nonprioritized substances REACH will not provide data for a meaningful hazard assessment of any end point.

### b) Increase data requirements

Even if a substantial number of chemicals in the lowest tonnage band will be prioritized, and consequently tested according to the REACH requirements, many substances produced or imported in quantities of < 10 metric tons/year will still lack the data necessary for a meaningful hazard assessment and classification according to the classification and labeling directive. It is clearly not in accordance with the objective of REACH to “ensure a high level of protection of human health and the environment” (European Commission 2006) to allow a large number of substances to be continuously put on the market and used when the minimal data required to assess their hazardous properties and classify and label them are not available. In our view, a decision should be made as soon as possible that after the last time limit for data requirements that has already been decided (year 2018), data requirements similar to those currently required for substances produced or imported in quantities of ≥ 10 metric tons/year will be introduced for all substances produced or imported in quantities of 1–10 metric tons yearly. Only then are we approaching a situation when the principle “no data, no market” can be said to have been realized. At the same time, a thorough evaluation needs to be made of the exclusion of substances produced or imported in quantities of < 1 metric ton/year from the legislation, and ways to include at least some of these substances into the system should be considered. These efforts should be combined with the work to develop new approaches to priority setting, data generation, and management of remaining uncertainties.

### c) Develop the tests and approaches needed to satisfy the information requirements

Before REACH went into force, it was estimated that 30,000 substances would be registered in the legislation. With the preregistration now completed, it includes some 145,000 substance—about five times as many as expected. To design appropriate test requirements for such a large and diverse group of substances in a resource-efficient and scientifically robust way is a difficult undertaking. The REACH process has triggered extensive discussions about how testing should effectively be performed in the regulatory context (e.g., Schaafsma et al. 2009; Scheringer et al. 2006; Rudén and Hansson 2007). How should chemicals be prioritized for testing? How extensive testing should be required? What (types of) tests should be included at different tiers? There is an urgent need for test systems that can fill the enormous data gap for insufficiently tested substances as efficiently as possible. Such a strategy must take into account the limitations in economic resources and testing capacity, and it must consider animal welfare and the aim to refine, reduce, and replace animal tests in regulatory toxicology. Furthermore, there is a desire to continuously incorporate novel toxicologic knowledge. A process to improve regulatory testing and risk assessment must therefore include new approaches and nontest methods [e.g., (Q)SAR, grouping, and read-across methods], development and standardization of novel test methods, and the determination of criteria and principles for risk assessment that can cope with new types of extrapolations and uncertainties.

### d) Promote substitution of high-risk chemicals

To manage chemical risks, we need to refrain from using chemicals in ways that give rise to unacceptable risks. To avoid this, we need to substitute high-risk substances with less risky alternatives as much as possible. In REACH the most hazardous substances (“substances of very high concern”) are identified as those that are classified as carcinogenic, toxic to reproduction or mutagenic in category 1 or 2, PBT or vPvB, or endocrine disrupting or give rise to an “equivalent level of concern” (REACH article 57). Such substances are candidates for inclusion in the list of substances subject to authorization (REACH annex XIV). However, as we have shown above, the REACH data requirements are in general insufficient for identifying chemicals with these properties. So far, fewer than 20 substances have been identified as substances of very high concern within REACH (see the European Chemicals Agency 2009), possibly because the legislation only recently came into force, but concerns have been raised that the regulatory process of identifying substances of very high concern will be slow and bureaucratic [see, e.g., the Substitute It Now (SIN) Reporter; International Chemical Secretariat 2009]. To achieve the goals of REACH, it is essential to develop an efficient process for identifying substances of very high concern and making the appropriate risk management decisions for these compounds.

### e) Acknowledge uncertainties

The introduction of REACH does not change the fact that the regulatory system (including the classification and labeling criteria in EEC 1967) does not distinguish between a substance that has been tested with negative outcome (no harmful effect detected) and a substance that has not been tested at all. In both these cases, the substance will be treated as having low or no toxicity. This is a risk-prone way of managing chemicals that needs to be replaced by a system of risk management that takes into account not only known harmful effects but also uncertainties. The classification and labeling system should be modified to include reports of lack of data. We propose the introduction of a labeling symbol to be used when basic toxicity information about a substance is lacking (Hansson and Rudén 2003). Significant improvements in the reporting of uncertainties are also needed in safety data sheets.

### f) Develop rules for chemicals in articles

Although REACH focuses on chemical substances and products, significant exposures of both humans and the environment are mediated by items that include or have been treated with chemicals. To take just one example, we are exposed to brominated flame retardants through electronic devices, furniture, building materials, and so on, rather than through chemical products. Tracing chemical substances in articles such as these is admittedly a much larger undertaking than that of keeping track of the contents of chemical products. Probably, a less comprehensive system will have to be chosen for articles than for chemical products. Nevertheless, a strategy for the risk management of chemicals is severely incomplete unless it tackles the distribution of harmful substances through the wide varieties of products in which they are used.

In this commentary we have shown that the outcome of the testing required in REACH is more uncertain than what may be generally realized. Important decisions remain to be made, particularly on triggers for testing of low-volume substances and on practices concerning the waiving of testing for substances in the high tonnage bands. The aim of REACH is to “ensure a high level of protection of human health and the environment.” With this commentary we hope to contribute to a scientific discussion about these open issues for the future development of REACH.

## Figures and Tables

**Table 1:** REACH data requirements: ecotoxicity.

	Metric tons/year				
Category	< 1	≥ 1	≥ 10	≥ 100	≥ 1,000
Long-term or reproductive toxicity: birds	No	No	No	No	(Yes?)
Long-term toxicity sediment: organisms	No	No	No	No	(Yes?)
Long-term toxicity: plants and terrestrial organisms	No	No	No	No	(Yes?)
Short-term toxicity: plants and terrestrial organisms	No	No	No	(Yes?)	(Yes?)
Long-term toxicity: fish and *Daphnia*^a^	No	No	No	(Yes?)	(Yes?)
Activated sludge respiration inhibition	No	No	Yes	Yes	Yes
Short-term toxicity: fish^a^	No	No	Yes	Yes	Yes
Short-term toxicity: algae^a^	No	(No?)	Yes	Yes	Yes
Short-term toxicity: *Daphnia*^a^	No	(No?)	Yes	Yes	Yes

(No?): testing can be triggered according to certain criteria; (Yes?): testing can be waived according to certain criteria.

a The test has a direct use for classification purposes.

**Table 2:** REACH data requirements: fate and behavior.

	Metric tons/year				
Category	< 1	≥ 1	≥ 10	≥ 100	≥ 1,000
Bioaccumulation in fish^a^	No	No	No	(Yes?)	(Yes?)
Identification of degradation products	No	No	No	(Yes?)	(Yes?)
Simulation testing^a^	No	No	No	(Yes?)	(Yes?)
Hydrolysis	No	No	Yes	Yes	Yes
Adsorption/desorption screening	No	No	Yes	Yes	Yes
Biotic degradation (ready biodegradation)^a^	No	(No?)	Yes	Yes	Yes

(No?): testing can be triggered according to certain criteria; (Yes?): testing can be waived according to certain criteria.

a The test has a direct use for classification purposes.

**Table 3:** REACH data requirements: toxicity.

	Metric tons/year				
Category	< 1	≥ 1	≥ 10	≥ 100	≥ 1,000
Chronic toxicity and carcinogenicity^a^	No	No	No	No	(Yes?)
Reproductive toxicity (one generation)^a^	No	No	No	(Yes?)	(Yes?)
Subchronic (90 days)^a^	No	No	No	(Yes?)	(Yes?)
Screening for reproductive toxicity	No	No	(Yes?)	(Yes?)	(Yes?)
Subacute (28 days)^a^	No	No	(Yes?)	Yes	Yes
Acute toxicity second route^a^	No	No	Yes	Yes	Yes
Skin + eye irritation (*in vivo*)^a^	No	No	Yes	Yes	Yes
Additional mutagenicity tests (*in vitro*)	No	No	Yes	Yes	Yes
Acute toxicity oral route^a^	No	(No?)	Yes	Yes	Yes
Mutagenicity (*in vitro*)	No	(No?)	Yes	Yes	Yes
Skin sensitization^a^	No	(No?)	Yes	Yes	Yes
Skin + eye irritation (*in vitro*)	No	(No?)	Yes	Yes	Yes

(No?): testing can be triggered according to certain criteria; (Yes?): testing can be waived according to certain criteria.

a The test has a direct use for classification purposes.
